# Real-World Phenotypic Profiles and Longitudinal Lung Function Outcomes in Severe Asthma Treated with Biologic Therapies

**DOI:** 10.3390/jpm16070362

**Published:** 2026-07-03

**Authors:** Ourania S. Kotsiou, Georgios I. Barkas, Konstantinos I. Gourgoulianis, Zoe Daniil

**Affiliations:** 1 Laboratory of Human Pathophysiology, Department of Nursing, University of Thessaly, Gaiopolis, 41500 Larissa, Greece; 2 Department of Respiratory Medicine, University of Thessaly, Biopolis, 41110 Larissa, Greece

**Keywords:** severe asthma, biologic therapy, real-world study, asthma phenotypes, lung function, small airways dysfunction, eosinophilic asthma, precision medicine

## Abstract

**Background:** Biologic therapies have transformed severe asthma management, but real-world evidence comparing phenotypes, lung function trajectories, and persistence across biologic classes remains limited. **Objective:** To characterize a real-world cohort of biologic-treated severe asthma patients, focusing on baseline phenotypes, longitudinal post-bronchodilator spirometry (including a spirometric surrogate suggestive of small airways involvement), and discontinuation/switching patterns. **Methods:** In this retrospective observational study at a tertiary referral center, adults with severe asthma treated with benralizumab, mepolizumab, omalizumab, or tezepelumab were included. Demographic, clinical, biomarker, and functional data were collected at baseline and follow-up. Post-bronchodilator FEV_1_ and FEF25–75 (% predicted) were assessed at baseline, 6 months, 12 months, and 24–36 months when available. Longitudinal outcomes were analyzed using multivariable linear mixed-effects models; discontinuation and switching were recorded. **Results:** Eighty-seven patients were included (benralizumab *n* = 13, omalizumab *n* = 10, mepolizumab *n* = 30, tezepelumab *n* = 34), representing 10.9% of the clinic’s population. Most had long-standing disease, elevated body mass index, and a T2-high profile. Baseline characteristics were generally similar across groups, with expected differences in total IgE (*p* = 0.007) and blood eosinophils (*p* < 0.001). The primary endpoint (FEV_1_ % predicted change from baseline to 12 months) showed adjusted mean changes of +12.46 (95% CI +1.63 to +19.29; *p* = 0.020) with benralizumab, +15.82 (+8.35 to +23.64; *p* < 0.001) with mepolizumab, +16.65 (+1.58 to +31.71; *p* < 0.001) with omalizumab, and +15.69 (+6.52 to +24.87; *p* = 0.030) with tezepelumab; trajectories differed by biologic class (time × biologic *p* = 0.019). Although the interaction term indicated heterogeneous temporal patterns, these adjusted findings should be interpreted as associative in the context of biomarker-driven treatment selection and not as evidence of comparative superiority of any biologic class. Discontinuation occurred in 15/87 (17.2%), with switching most commonly due to inadequate control. **Conclusions:** Real-world severe asthma patients demonstrate heterogeneous phenotypes and spirometric trajectories on biologics. Integrating biomarkers with longitudinal lung function monitoring, including small-airway spirometric surrogates, supports individualized management.

## 1. Introduction

Severe asthma represents a heterogeneous and clinically challenging condition, accounting for a disproportionate burden of morbidity and healthcare utilization despite affecting a minority of patients with asthma [1,2]. It is characterized by persistent symptoms and frequent exacerbations despite optimized high-dose inhaled corticosteroid therapy and additional controllers [1].

Advances in the understanding of asthma pathophysiology have led to the identification of distinct inflammatory endotypes, particularly type 2 (T2)-high and T2-low disease, enabling the development of targeted biologic therapies [3,4]. Biologic agents targeting IgE, interleukin (IL)-5, the IL-5 receptor, and upstream epithelial cytokines have demonstrated efficacy in reducing exacerbations, improving asthma control, and decreasing oral corticosteroid dependence in selected populations [5,6,7,8,9].

Although randomized controlled trials have established the efficacy of biologics, real-world studies highlight substantial heterogeneity in treatment response and patient characteristics, underscoring the need for observational data from routine clinical practice [10,11,12,13,14,15,16,17]. Comparative real-world data evaluating lung function trajectories, treatment persistence, and switching patterns across different biologic classes remain limited [18].

Small airways involvement is increasingly recognized as a clinically relevant feature of severe asthma, often persisting despite relatively preserved FEV_1_ and contributing to symptoms, airflow limitation, and disease severity [13,14]. In this context, FEF25–75 may serve as a spirometric surrogate suggestive of small airways involvement, providing complementary information to conventional spirometric indices, although it is not a gold-standard measure and should be interpreted with caution.

The present study aimed to characterize real-world phenotypic profiles and longitudinal spirometric trajectories in patients with severe asthma receiving biologic therapies. Because treatment assignment in routine care is non-random and largely guided by biomarkers and clinical features, we framed the work as a descriptive, covariate-adjusted longitudinal assessment rather than a comparative effectiveness analysis. We therefore (i) summarized phenotypic characteristics by biologic class, (ii) modeled within-patient changes over time in conventional and small-airway spirometric indices, and (iii) described treatment persistence, discontinuation, and switching patterns in clinical practice, without inferring causality or claiming between-biologic superiority.

## 2. Methods

### 2.1. Study Design and Setting

This was a real-world, observational study conducted at the Asthma Clinic of the Department of Respiratory Medicine, University of Thessaly, Larissa, Greece. The clinic is a tertiary referral center for patients with severe asthma and provides longitudinal follow-up and biologic therapy according to international guidelines. Data were collected retrospectively from routinely recorded clinical and laboratory records.

### 2.2. Study Population

The study population comprised adults with severe asthma who received biologic therapy as part of routine clinical care. The External Asthma Unit follows approximately 800 patients annually; during 2021–2025, every patient treated with a biologic was eligible and included. A formal sample-size calculation was not undertaken because enrollment was exhaustive for the study period.

Severe asthma was defined according to ERS/ATS and GINA criteria as asthma requiring high-dose inhaled corticosteroids (ICS) plus a second controller (long-acting β_2_-agonist [LABA]) and/or systemic corticosteroids to maintain control or remaining uncontrolled despite this regimen [2]. Severity at biologic initiation was additionally profiled using standardized clinical descriptors beyond spirometry, including maintenance oral corticosteroid (OCS) use and daily OCS dose. Asthma control was measured with the Asthma Control Test (ACT; range 5–25), with scores < 20 indicating uncontrolled disease.

At initiation of biologic therapy, all patients were receiving GINA Step 5 treatment with high-dose ICS/LABA, with optional add-on controllers such as long-acting muscarinic antagonists (LAMA), leukotriene receptor antagonists (LTRA), or theophylline. Comorbidities were systematically recorded, including allergic rhinitis, chronic rhinosinusitis with nasal polyposis, gastroesophageal reflux disease (GERD), obesity, and atopic dermatitis.

Biologic prescribing followed national reimbursement requirements and ERS/ATS and GINA guidance. Anti-IL-5/IL-5R agents were used for eosinophilic asthma with recurrent exacerbations and/or OCS dependence; omalizumab for allergic asthma with IgE levels within approved dosing ranges; and tezepelumab for severe asthma remaining inadequately controlled despite optimized therapy, including patients with lower conventional T2 biomarker levels [1,2,3].

### 2.3. Ethical Considerations

The study was conducted in accordance with the Declaration of Helsinki and received retroactive approval by the Scientific Council of the University General Hospital of Larissa (Approval No. 15685, 2 April 2026; additional clarifying statement: Ref. No. 29033/19-06-2026, Decision No. 32/12th, 16 June 2026, Regular Meeting of the Scientific Council).

Written informed consent was obtained from all participants for the use of their data in the present study.

### 2.4. Study Outcomes

The primary endpoint of the study was the change in post-bronchodilator FEV_1_ % predicted from baseline to 12 months after initiation of biologic therapy.

Secondary endpoints included (i) change in post-bronchodilator FEF25–75% predicted from baseline to 12 months; (ii) longitudinal trajectories of FEV_1_ and FEF25–75 across all available time points (baseline, 6 months, 12 months, and 24–36 months); (iii) treatment persistence; (iv) annualized exacerbation outcomes during follow-up; and (v) discontinuation and switching patterns.

### 2.5. Data Collection and Variables

Demographic data included age, sex, body mass index (BMI), smoking status, and asthma duration. Clinical variables comprised asthma phenotype, comorbid allergic rhinitis, nasal polyposis, eczema, and maintenance OCS use at biologic initiation.

Baseline inflammatory assessment included total serum immunoglobulin E (IgE), blood eosinophil count, and fractional exhaled nitric oxide (FeNO), when available. Type 2 (T2) inflammatory status was assigned before biologic initiation using a combined biomarker–clinical framework. Patients were categorized as T2-high if any of the following were present: blood eosinophils ≥ 150 cells/μL at baseline or ≥300 cells/μL within the previous 12 months; FeNO ≥ 25 ppb; evidence of allergic sensitization with elevated IgE and/or allergic asthma; or T2-associated comorbidities (allergic rhinitis, nasal polyposis, eczema). T2-low asthma was defined by absence of all these features.

When biomarker data were incomplete, classification relied on the available markers together with the overall clinical phenotype, reflecting standard practice in specialized severe asthma services and consistent with ERS/ATS and GINA guidance and prior real-world reports [1,2,3,4].

### 2.6. Lung Function Assessment

Spirometry was performed using a Spirolab spirometer (MIR—Medical International Research S.p.A., Rome, Italy). It was conducted by trained personnel following standardized procedures, with the same equipment and calibration routines used throughout the study period to support measurement consistency. Baseline post-bronchodilator parameters (ATS/ERS standards) included FEV_1_, FEV_1_ (% predicted), forced vital capacity (FVC), FEV_1_/FVC ratio, and forced expiratory flow between 25% and 75% of FVC (FEF25–75).

FEF25–75 was analyzed as a pragmatic spirometric proxy for small airways involvement. We recognize its limitations, including effort dependence, sensitivity to changes in FVC, and the fact that it is not a gold-standard measure of small-airways disease. More specialized assessments (e.g., impulse oscillometry, multiple-breath nitrogen washout, RV/TLC, or CT-based air trapping) were not systematically available in this retrospective real-world cohort.

Longitudinal spirometry was evaluated at baseline (pre-biologic), 6 months, 12 months, and 24–36 months after initiation as part of routine follow-up. Both absolute and percent predicted values were examined. The 24–36-month interval was defined as any assessment performed between 24 and 36 months; when more than one test fell within a window, the measurement closest to the nominal time point was retained. Follow-up duration was calculated from biologic initiation to the last recorded clinical assessment.

All patients had baseline spirometry, and follow-up completeness is detailed in Supplementary Tables S1 and S2. As expected in routine care, the number of available spirometric observations declined over time, reflecting variable follow-up and discontinuation of the index biologic (15/87; 17.2% overall). Patients often continued spirometric monitoring clinically, but analyses were restricted to measurements occurring within the predefined index-biologic follow-up windows. Tezepelumab became available in Greece in September 2024, accounting for the lack of 24–36-month data in that subgroup.

### 2.7. Healthcare Utilization and Treatment Outcomes

Healthcare utilization outcomes comprised asthma-related emergency department visits and hospitalizations during the 12 months before biologic initiation.

Exacerbations were captured during follow-up and defined as episodes requiring systemic corticosteroids and/or an emergency department visit or hospitalization. Exacerbation follow-up was aligned with the duration of exposure to the index biologic.

Treatment-related outcomes included permanent discontinuation of the index biologic, clinician-documented reasons for discontinuation (inadequate control, loss of response, non-adherence, adverse events), and subsequent switching patterns. Treatment persistence was defined as the interval from biologic initiation to permanent discontinuation. Discontinuation referred to definitive cessation for any of the reasons above (or other documented clinical reasons). Temporary interruption was defined as a treatment gap followed by re-initiation of the same agent and was not counted as discontinuation. Switching was defined as initiation of a different biologic after stopping the index biologic.

Patients who remained on the index biologic at last follow-up were censored at the date of the most recent clinic visit or biologic administration. Those lost to follow-up without documented discontinuation were censored at the date of last clinical contact.

Persistence was evaluated using Kaplan–Meier methods, with between-group differences assessed using the log-rank test. Given the observational, non-randomized design and phenotype/biomarker-driven treatment allocation, any between-biologic comparisons were interpreted descriptively rather than as evidence of comparative effectiveness.

### 2.8. Statistical Analysis

Continuous variables are summarized as mean ± standard deviation (SD) or median (interquartile range [IQR]), as appropriate, and categorical variables as counts and percentages. Baseline differences across biologic classes were assessed with one-way ANOVA for normally distributed continuous variables, the Kruskal–Wallis test for non-normally distributed continuous variables, and the χ^2^ test (or Fisher’s exact test when expected cell counts were small) for categorical variables. All tests were two-sided, with *p* < 0.05 considered statistically significant unless otherwise stated.

Longitudinal lung function was evaluated using multivariable linear mixed-effects models with patient-specific random intercepts to account for within-subject correlation across repeated measures. Time since biologic initiation was specified as a categorical fixed effect (baseline, 6 months, 12 months, and 24–36 months). Fixed effects included time, biologic class, and prespecified covariates. To reduce confounding by indication, models were adjusted a priori for age, sex, body mass index, smoking status, baseline FEV_1_ (% predicted), baseline blood eosinophil count, asthma duration, and maintenance OCS use at initiation. In secondary models, time × biologic class interactions were added to explore potential heterogeneity in trajectories across therapies.

Models were estimated using restricted maximum likelihood (REML), assuming normally distributed random effects. Assumptions were checked using residual diagnostics. Results are reported as β estimates (adjusted mean change) with 95% confidence intervals and exact two-sided *p*-values. Mixed-effects models were fit under a missing-at-random (MAR) framework, incorporating all available observations without listwise deletion.

The prespecified primary endpoint was the change in post-bronchodilator FEV_1_ (% predicted) from baseline to 12 months. Secondary endpoints included change in FEF25–75 (% predicted), trajectories across all time points, treatment persistence, discontinuation and switching patterns, and exacerbation outcomes. To limit type I error, confirmatory inference was restricted to the primary endpoint (two-sided α = 0.05); all secondary analyses were considered exploratory.

Sensitivity analyses comprised (i) complete-case analyses including only patients with both baseline and 12-month spirometry and (ii) analyses restricted to T2-high patients, additionally applying a stricter eosinophil threshold (≥300 cells/μL), to enhance subgroup comparability and test robustness. Persistence was examined with Kaplan–Meier methods and the log-rank test; given phenotype-driven treatment selection, between-group persistence comparisons were interpreted descriptively.

All analyses were performed using IBM SPSS Statistics for Windows, version 25.0 (IBM Corp., Armonk, NY, USA). The study is reported in accordance with STROBE guidelines (Supplementary File S1).

## 3. Results

### 3.1. Study Population and Data Completeness

From an annual population of 800 patients followed at the clinic, 87 patients (10.9%) were treated with biologic agents and included in the analysis. The study population comprised patients receiving benralizumab (n = 13), omalizumab (n = 10), mepolizumab (n = 30), and tezepelumab (n = 34).

### 3.2. Baseline Characteristics of the Overall Biologic-Treated Population

The mean age of the overall cohort was 61 ± 9 years. Overall, 65/87 (74.7%) were never-smokers and 22/87 (25.3%) were current or former smokers. BMI was elevated across treatment groups (Table 1), indicating a predominantly overweight/obese population. Overall, 72/87 patients (82.8%) were classified as T2-high and 15/87 (17.2%) as T2-low; notably, T2-low patients were observed primarily in the tezepelumab group (Table 1).

### 3.3. Comorbidities, Corticosteroid Use, and Biomarkers

At biologic initiation, asthma control was poor in most patients. Among those with available data, the mean Asthma Control Test (ACT) score was 15.8 ± 4.6, reflecting predominantly uncontrolled disease. Overall, 61/87 (70.1%) had ACT < 20, whereas 26/87 (29.9%) had ACT ≥ 20, indicating partially controlled or controlled asthma.

Allergic rhinitis was frequent (60/87; 69.0%), with nasal polyps documented in 26/87 (29.9%) and atopic dermatitis in 17/87 (19.5%). Chronic or intermittent OCS use at initiation was recorded in 30/87 (34.5%). All patients were receiving GINA Step 5 therapy with high-dose ICS/LABA plus additional controllers, as indicated.

Baseline biomarker profiles varied across biologic classes, consistent with biomarker-guided prescribing. Total IgE was highest in the omalizumab group and lowest in the tezepelumab group (Table 1; *p* = 0.007). Blood eosinophils were greatest among anti-IL-5/IL-5R recipients (mepolizumab: 541 ± 311 cells/μL; benralizumab: 503 ± 592 cells/μL) and lower in the omalizumab and tezepelumab groups (203 ± 303 and 181 ± 167 cells/μL, respectively; Table 1; *p* < 0.001). FeNO values were similar across groups (Table 1).

### 3.4. Lung Function, Healthcare Utilization, and Treatment Discontinuation

Baseline lung function reflected persistent airflow limitation despite optimized therapy. The mean FEV_1_ was 2.45 ± 0.75 L, corresponding to 90 ± 18% predicted, while the mean FEV_1_/FVC ratio was 76 ± 9%. Mean FEF25–75 (% predicted) did not differ significantly between biologic groups, indicating broadly comparable average mid-expiratory flow impairment across treatments at baseline.

In the 12 months preceding biologic initiation, patients experienced a substantial exacerbation burden and frequent healthcare utilization. The mean annualized exacerbation rate was 1.8 ± 1.2 events per patient-year, and exacerbations requiring systemic corticosteroids occurred at a rate of 1.4 ± 1.0 events per patient-year. Asthma-related emergency department visits averaged 0.42 ± 0.66 events per patient-year, while hospitalizations occurred at a rate of 0.26 ± 0.51 events per patient-year, reflecting clinically significant disease activity despite optimized standard therapy (Supplementary Table S3).

### 3.5. Baseline Comparison Between Biologic Treatment Groups

Female patients predominated across all biologic groups: benralizumab 10/13 (76.9%), omalizumab 8/10 (75.0%), mepolizumab 23/30 (75.0%), and tezepelumab 26/34 (75.9%). Mean age differed numerically between groups but not significantly (Table 1; *p* = 0.287). BMI was high across all groups without statistically significant differences (Table 1; *p* = 0.282).

Baseline lung function indices (FEV_1_ % predicted, FEV_1_/FVC, and FEF25–75% predicted) were broadly similar across biologic groups (all *p* > 0.05) (Table 1). In contrast, biomarkers differed as expected given real-world biologic selection: IgE levels varied significantly (*p* = 0.007), and eosinophil counts differed substantially, with highest values in anti-IL-5/IL-5R groups (*p* < 0.001) (Table 1).

T2-high phenotype was present in all patients receiving benralizumab, omalizumab, and mepolizumab (100% in each group), whereas the tezepelumab group included both T2-high and T2-low patients (19/34, 55.6% T2-high; Table 1). The between-group difference reached statistical significance.

Overall, these baseline patterns reflect routine clinical prescribing in which biologic choice aligns with dominant inflammatory traits and disease history rather than random allocation.

### 3.6. Longitudinal Spirometric Outcomes Across Biologic Treatment Groups

Post-bronchodilator spirometry was available for all patients at baseline and at 6 months (87/87), for 77/87 patients at 12 months, and for 42/87 patients at 24–36 months (Supplementary Table S1). By biologic group, spirometric availability at 24–36 months was 11/13 for benralizumab, 8/10 for omalizumab, 23/30 for mepolizumab, and 0/34 for tezepelumab. Discontinuation of the index biologic occurred in 15/87 (17.2%) patients overall. Patients continued to be followed spirometrically in routine care; however, longitudinal analyses are presented according to the availability of measurements within the index-biologic follow-up window.

In the adjusted mixed-effects model, the time × biologic class interaction was statistically significant (Wald χ^2^(6) = 15.12, *p* = 0.019), indicating heterogeneity in FEV_1_ trajectories across biologic groups after accounting for baseline clinical differences. The adjusted mean change in FEV_1_ (% predicted) from baseline to 12 months was +12.46 (95% CI +1.63 to +19.29; *p* = 0.020) for benralizumab, +15.82 (95% CI +8.35 to +23.64; *p* < 0.001) for mepolizumab, +16.65 (95% CI +1.58 to +31.71; *p* < 0.001) for omalizumab, and +15.69 (95% CI +6.52 to +24.87; *p* = 0.030) for tezepelumab. Adjusted estimates of lung function change derived from multivariable mixed-effects models are presented in Table 2.

Despite evidence of heterogeneity in temporal patterns, these adjusted estimates should be interpreted as associations within a biomarker-guided, non-randomized setting rather than as evidence of comparative superiority. Overall, lung function was maintained or improved over time across biologic therapies at the population level.

**Figure 1:**
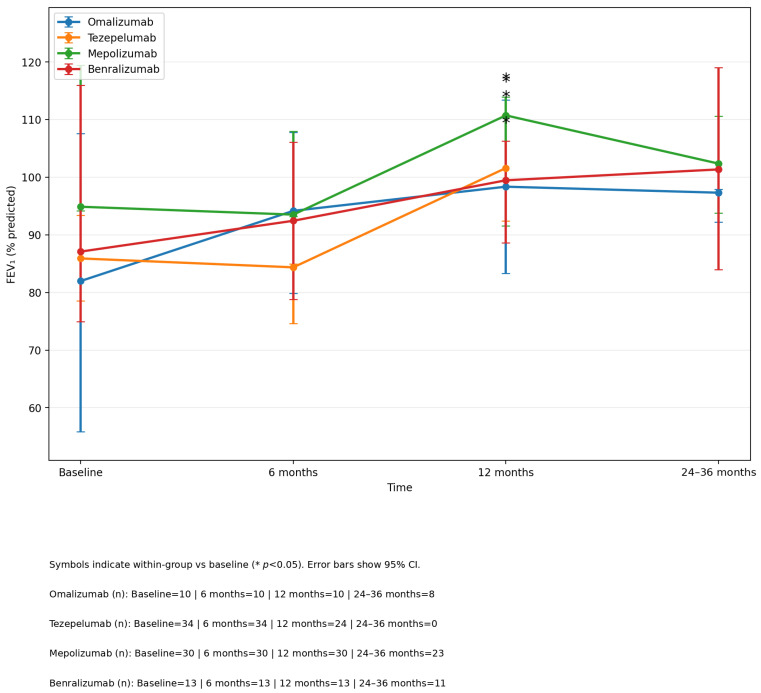
Mean FEV1 (% predicted) values are shown at baseline, 6 months, 12 months, and 24–36 months for patients treated with omalizumab, mepolizumab, benralizumab, and tezepelumab. Error bars represent 95% confidence intervals. The number of patients contributing data at each time point is shown below the x-axis for each biologic group. Statistical symbols indicate within-group comparisons versus baseline (* *p* < 0.05), and between-group comparisons are reported in the figure annotation/caption. The number of patients at each time point reflects available follow-up measurements and decreases over time due to biologic discontinuation and unequal follow-up duration.

**Figure 2:**
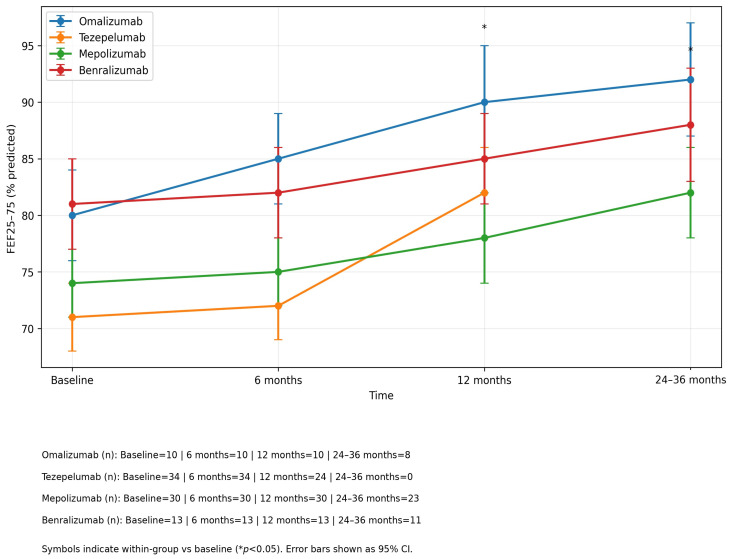
Mean post-bronchodilator FEF25–75 (% predicted), used here as a spirometric surrogate suggestive of small airways involvement, is shown at baseline, 6 months, 12 months, and 24–36 months after biologic initiation for omalizumab, mepolizumab, and benralizumab. The 24–36-month assessments were unavailable for the tezepelumab group. Error bars represent 95% confidence intervals. The number of patients contributing data at each time point is provided below the x-axis for each biologic group. Statistical symbols indicate within-group comparisons versus baseline (* *p* < 0.05). The number of patients at each time point reflects available follow-up measurements and decreases over time due to biologic discontinuation and unequal follow-up duration.

FEV1 was assessed spirometrically at each scheduled follow-up visit; however, the number of patients contributing data at later time points differs because some patients discontinued the index biologic during follow-up (15/87, 17.2%). Patients were still followed spirometrically in routine care, but analyses are presented according to availability of measurements under the index-biologic follow-up window (Figure 1 and Figure 2).

### 3.7. Longitudinal Outcomes in Patients with Severe Asthma Treated with Biologic Therapies

Following biologic initiation, significant improvements were observed across all exacerbation-related outcomes (Supplementary Table S3). The annualized exacerbation rate decreased to 0.7 ± 0.9 events per patient-year, corresponding to a mean reduction of −1.1 events per patient-year (95% CI: −1.4 to −0.8; *p* < 0.001). Exacerbations requiring systemic corticosteroids declined to 0.5 ± 0.7 events per patient-year (mean difference −0.9; 95% CI: −1.2 to −0.6; *p* < 0.001). Emergency department visits and hospitalizations were also significantly reduced to 0.15 ± 0.38 and 0.08 ± 0.29 events per patient-year, respectively (both *p* < 0.01).

Overall, biologic therapy was associated with reductions in exacerbation frequency, systemic corticosteroid-treated exacerbations, emergency department visits, and hospitalizations during follow-up in this real-world cohort.

To evaluate the robustness of the primary findings under a stricter biomarker-based definition of T2-high disease, we performed a sensitivity analysis restricted to patients with baseline blood eosinophil counts ≥ 300 cells/μL. A total of 32/87 patients met this criterion, including benralizumab (n = 6), omalizumab (n = 3), mepolizumab (n = 19), and tezepelumab (n = 4) (Supplementary Table S4).

In this subgroup, paired baseline and 12-month post-bronchodilator FEV_1_ (% predicted) data were available for all 32 patients. The direction of lung function change was consistent with the primary analysis, supporting the overall pattern of stabilization/improvement after biologic initiation in biomarker-enriched patients. Given the reduced subgroup size—particularly within omalizumab and tezepelumab—this sensitivity analysis was interpreted as supportive and exploratory rather than confirmatory.

### 3.8. Phenotypic Characterization of Biologic-Treated Patient Groups

Patients treated with benralizumab represented a predominantly female, middle-aged population with long-standing severe asthma and a T2-high inflammatory phenotype. This group demonstrated high blood eosinophil counts and a high prevalence of atopic comorbidities. Baseline spirometry showed airflow limitation with frequent evidence of reduced mid-expiratory flow (FEF25–75), used here as a spirometric surrogate suggestive of small airways involvement. Longitudinally, spirometric indices showed numerical improvement and stabilization over follow-up.

Patients receiving mepolizumab constituted a large cohort characterized by eosinophilic inflammation, with the highest baseline blood eosinophil levels among all biologic groups. Disease chronicity and corticosteroid exposure were common, and baseline spirometry demonstrated airflow obstruction with reduced FEF25–75 values, suggestive of small airways involvement. Over time, lung function changes were characterized by stabilization and significant numerical improvement.

The omalizumab group reflected a predominantly allergic severe asthma phenotype with higher total IgE and relatively lower eosinophil counts than anti-IL-5/IL-5R groups. Baseline lung function was relatively preserved, and reduced FEF25–75 values (spirometric surrogate suggestive of small airways involvement) were as prominent as in other groups. Longitudinally, spirometric indices showed improvement and maintenance over time.

Patients treated with tezepelumab formed the most heterogeneous group, including both T2-high and T2-low phenotypes. This cohort was older and demonstrated a broad range of biomarker profiles. Baseline spirometry frequently showed airflow limitation and reduced FEF25–75 values, suggestive of small airways involvement. Over follow-up, improvements in lung function were observed, with changes becoming evident by 12 months.

**Figure 3:**
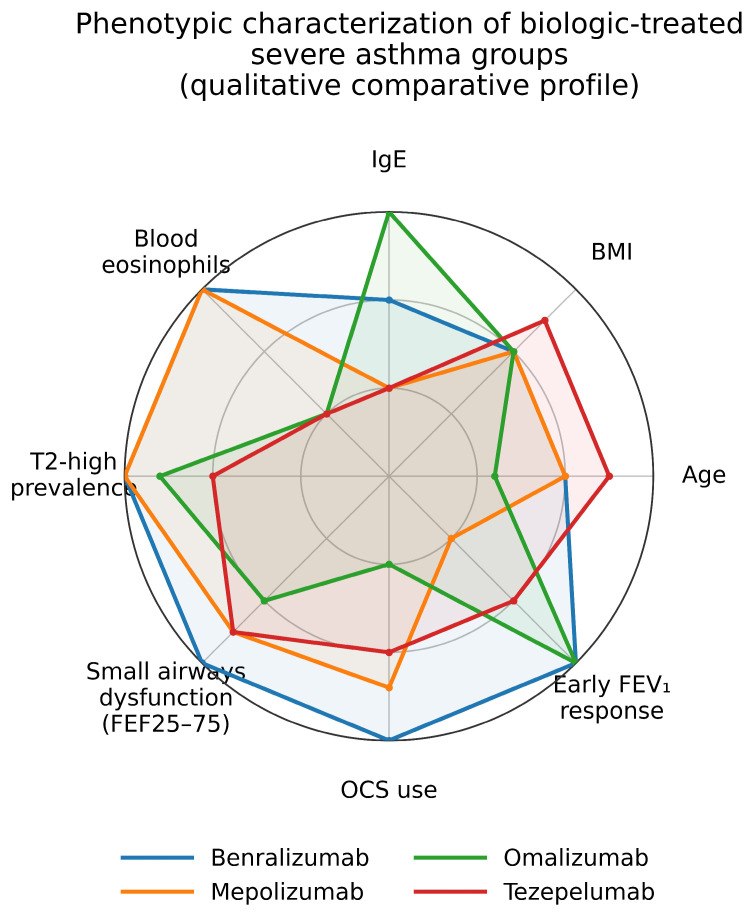
The radar plot illustrates demographic, inflammatory, and functional characteristics across biologic treatment groups. Colored shaded areas and corresponding line contours illustrate qualitative comparative profiles of biologic-treated severe asthma groups. Each axis represents one clinical or biological feature. Greater distance from the center indicates greater relative prominence of that feature. The figure is intended for visual comparison only and does not represent absolute quantitative values or statistical effect sizes.

Overall, phenotypic profiles across biologic groups reflect real-world precision medicine practice, where treatment selection aligns with dominant inflammatory traits and disease history rather than random assignment (Figure 3).

### 3.9. Treatment Discontinuation and Persistence

During follow-up, permanent discontinuation of the index biologic occurred in 15/87 (17.2%) patients overall. By biologic group, discontinuation occurred in 2/13 (15.4%) patients treated with benralizumab, 2/10 (20.0%) treated with omalizumab, 7/30 (23.3%) treated with mepolizumab, and 4/34 (11.8%) treated with tezepelumab.

**Figure 4:**
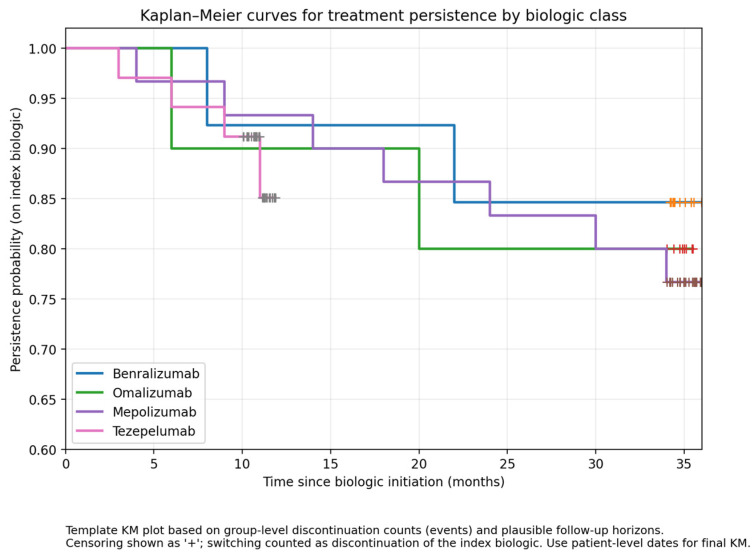
Kaplan–Meier curves for treatment persistence by biologic class. Footnote: Persistence was defined as time from biologic initiation to permanent discontinuation of the index biologic; switching was counted as discontinuation. Patients remaining on treatment were censored at last follow-up. Between-group comparisons are descriptive due to non-random, phenotype-driven treatment selection and unequal group sizes. The overlapping elements in the original figure concerned censoring marks occurring at similar late follow-up time points and did not affect the scientific interpretation of the Kaplan–Meier curves.

The most common reasons for discontinuation were inadequate asthma control and/or loss of response, followed by non-adherence or logistical reasons. Discontinuation due to adverse events was not reported. Kaplan–Meier analysis showed broadly comparable treatment persistence across biologic classes (log-rank *p* > 0.05), although interpretation is limited by subgroup size imbalance and phenotype-driven biologic selection (Figure 4).

### 3.10. Biologic Switching Patterns

Among patients who discontinued their initial biologic therapy, switching to an alternative biologic agent was the predominant strategy. Overall, 12/15 (80.0%) discontinuations resulted in a switch rather than permanent cessation of biologic treatment.

Among patients discontinuing mepolizumab (n = 7), 6/7 (85.7%) switched to another biologic agent. Of these, 4/6 (66.7%) transitioned to benralizumab, reflecting escalation within the anti-IL-5/IL-5R pathway, while 2/6 (33.3%) switched to tezepelumab in the setting of persistent symptoms despite eosinophil suppression.

All patients discontinuing omalizumab (2/2, 100%) switched to alternative biologic therapy, with 1/2 (50.0%) transitioning to benralizumab and 1/2 (50.0%) to tezepelumab, particularly in the presence of mixed or non-allergic inflammatory features.

Switching from benralizumab was less frequent; however, when discontinuation occurred (n = 2), all patients (2/2, 100%) transitioned to tezepelumab, reflecting persistent disease activity despite effective eosinophil depletion.

Among tezepelumab discontinuations (n = 4), 2/4 switched and 2/4 discontinued without subsequent biologic during follow-up.

Overall, switching patterns reflected real-world, biomarker-driven treatment optimization, with preferential transitions from IgE-targeted and IL-5-targeted therapies toward IL-5R blockade or upstream epithelial inhibition, rather than lateral switching within the same biologic class.

## 4. Discussion

In this real-world observational study conducted in a tertiary severe asthma clinic, we provide a detailed characterization of adults treated with benralizumab, mepolizumab, omalizumab, or tezepelumab, integrating baseline phenotypic features with longitudinal spirometric trajectories, a pragmatic small-airways surrogate, healthcare utilization, and treatment persistence and switching patterns. Collectively, the findings emphasize the substantial heterogeneity of biologic-treated severe asthma in routine care and support the clinical necessity of combining inflammatory biomarkers with functional assessment and longitudinal monitoring to inform precision-oriented decision-making.

The overall biologic-treated population was characterized by long-standing disease, elevated body mass index, and a predominance of T2-high inflammatory phenotypes, consistent with previous epidemiological and real-world studies conducted in specialized severe asthma centers [1,10].

Baseline comparisons between biologic treatment groups revealed broadly comparable demographic and clinical characteristics, with no statistically significant differences in most variables. This pattern reflects real-world prescribing practices, in which biologic selection is primarily guided by dominant inflammatory traits and prior treatment response rather than by clearly separable severity strata [16,17,18]. Nevertheless, numerical differences in inflammatory biomarkers were observed, consistent with established mechanisms of action and eligibility criteria. As expected, anti-IL-5/IL-5R therapies were preferentially prescribed to patients with higher eosinophil burden, while omalizumab was primarily used in patients with allergic-driven disease and elevated IgE levels [5,6,7]. Dupilumab is not available in Greece; consequently, it was not represented in this cohort.

Notably, despite optimized background therapy (GINA Step 5), baseline spirometry showed persistent airflow limitation in 13.8% of the cohort (FEV_1_/FVC < 70%) and reduced mid-expiratory flows in the tezepelumab subgroup (38.2% with FEF25–75 < 70% predicted), supporting frequent distal airway involvement even when conventional indices appear relatively preserved [13,14].

Baseline comparisons across biologic groups showed broadly comparable demographic and clinical characteristics, with expected biomarker differences consistent with mechanism-based eligibility and reimbursement criteria. Higher IgE levels in omalizumab recipients and greater eosinophil burden among anti-IL-5/IL-5R recipients support the internal coherence of biomarker-guided prescribing in routine practice. The tezepelumab subgroup included a substantial proportion of patients classified as T2-low, reflecting its positioning as an option for biomarker-diverse severe asthma. Dupilumab was not represented, consistent with its lack of availability in Greece during the study period and limiting direct inference regarding IL-4/IL-13 pathway inhibition in this setting.

Phenotypic characterization further illustrates the practical implementation of precision medicine in routine care. Anti-IL-5/IL-5R therapies were predominantly used in patients with eosinophilic, often steroid-dependent disease and features of airway remodeling, omalizumab in younger patients with allergic-driven and more reversible disease, and tezepelumab in older, heterogeneous populations with mixed or low traditional T2 biomarkers. Despite these differences, baseline disease severity remained high across all groups, underscoring the advanced and refractory nature of patients receiving biologic therapy in clinical practice.

Across biologic classes, lung function was generally stabilized or modestly improved over time. Although the time × biologic interaction was statistically significant in adjusted mixed-effects models—indicating heterogeneity in trajectories—adjusted estimates did not demonstrate consistent superiority of any single biologic class. In non-randomized real-world cohorts, where biologic assignment is driven by phenotype, biomarker profile, comorbidity burden, reimbursement constraints, and calendar-time availability, such heterogeneity is more plausibly explained by confounding by indication and residual differences in baseline disease biology than by comparative differences in treatment efficacy [5,6,7,11,12,18]. Accordingly, these longitudinal patterns should be interpreted as associative and hypothesis-generating rather than causal or comparative-effectiveness evidence.

Using FEF25–75 < 70% predicted as a spirometric surrogate suggestive of small airways involvement, approximately one-third of tezepelumab-treated patients met this threshold (13/34; 38.2%). This pattern is consistent with phenotype-driven prescribing in routine practice and suggests that tezepelumab may have been preferentially selected for patients with more prominent distal-airway dysfunction and biomarker-diverse disease [19]. Future prospective cohorts incorporating validated distal-airway assessments (e.g., impulse oscillometry, multiple-breath washout, RV/TLC, or CT air trapping) are warranted to confirm whether tezepelumab-treated patients represent a distinct small-airway-predominant phenotype and to clarify the clinical implications for treatment response and persistence.

Overall, group mean FEV_1_ was stable to modestly improved over time, with the largest increases generally observed at 12 months, followed by maintenance or slight attenuation at 24–36 months in groups with longer follow-up. This calendar-time constraint also complicates cross-biologic comparisons at later time points and underscores the importance of follow-up standardization in future observational evaluations.

Longitudinally, changes in FEF25–75 broadly paralleled FEV_1_ trajectories, suggesting that biologic therapy may confer distal airway benefits in some patients, albeit with variable timing and magnitude. Nevertheless, interpretation of FEF25–75 requires caution due to its dependence on expiratory effort and its susceptibility to FVC variability. The absence of systematic advanced small-airway assessment (e.g., impulse oscillometry, multiple-breath washout, RV/TLC, or CT air-trapping quantification) represents an important limitation and highlights a clear priority for future prospective studies seeking mechanistic insight into distal airway response to biologics.

In addition to longitudinal spirometric patterns, biologic initiation was associated with clinically meaningful improvements in exacerbation burden and healthcare utilization. The annualized exacerbation rate decreased substantially during follow-up, accompanied by parallel reductions in systemic corticosteroid-treated exacerbations and asthma-related emergency department visits and hospitalizations. These findings are consistent with the established real-world effectiveness of biologic therapies in severe asthma, where reductions in exacerbations and acute-care utilization are often more robust and clinically salient than changes in spirometric indices. From a service perspective, the magnitude of improvement observed in acute events is particularly relevant, as it reflects reduced disease instability and may translate into lower healthcare costs, reduced corticosteroid exposure, and improved quality of life. Nevertheless, interpretation should remain cautious. In observational cohorts, improvements in exacerbation-related outcomes may reflect not only treatment effects but also concurrent optimization of background therapy, improved adherence through specialist follow-up, regression to the mean in patients selected during periods of high activity, and changes in exposure time due to discontinuation. In the present study, exacerbation follow-up was aligned with exposure to the index biologic, which strengthens internal consistency; however, residual confounding and time-varying factors inherent to real-world management cannot be excluded.

To evaluate robustness under a stricter biomarker-defined T2-high framework, we performed a sensitivity analysis restricted to patients with baseline blood eosinophils ≥ 300 cells/μL. In this biomarker-enriched subgroup, the direction of lung function change remained consistent with the primary analysis, supporting the overall pattern of stabilization/improvement after biologic initiation in patients with prominent eosinophilic inflammation. While reassuring, the reduced sample size—particularly for omalizumab and tezepelumab—limits precision and precludes confident between-class inference. Accordingly, this sensitivity analysis should be viewed as supportive rather than confirmatory and highlights the need for larger multicenter datasets to evaluate effect heterogeneity across biologic mechanisms within biomarker-defined strata.

Treatment persistence was high in this cohort. Permanent discontinuation occurred in a minority of patients (15/87; 17.2%), with discontinuation rates that did not meaningfully differ between biologic groups. Discontinuation was primarily attributable to inadequate asthma control and/or loss of response, followed by non-adherence or logistical reasons; discontinuation due to adverse events was not observed. Kaplan–Meier analyses suggested broadly comparable persistence across biologic classes (log-rank *p* > 0.05); however, these comparisons are best interpreted descriptively given unequal subgroup sizes, phenotype-driven prescribing, and differential follow-up (including tezepelumab’s limited long-term observation window). Together, these findings align with real-world experience that lack of sufficient clinical benefit—rather than intolerance—is the predominant driver of biologic discontinuation in severe asthma services.

Switching was the predominant strategy following discontinuation (12/15; 80%), indicating iterative treatment optimization rather than cessation of biologic therapy. Switching patterns were clinically coherent and consistent with biomarker-guided escalation or pathway re-targeting: a substantial proportion of mepolizumab discontinuations transitioned to benralizumab (suggestive of escalation within IL-5 pathway blockade), whereas switches to tezepelumab occurred in contexts of persistent symptoms despite eosinophil-directed therapy. Omalizumab discontinuations transitioned to benralizumab or tezepelumab, potentially reflecting mixed phenotypes or evolving inflammatory characteristics. These observations reinforce the pragmatic role of serial reassessment—integrating symptoms, exacerbations, spirometry, biomarkers, and comorbidity control—in guiding switching decisions.

Obesity may influence asthma control, lung mechanics, and response to therapy; therefore, residual confounding related to adiposity may have contributed to between-patient variability in lung function trajectories despite adjustment for BMI [20]. We adjusted models for BMI but did not perform BMI-stratified analyses due to limited subgroup sizes; residual confounding related to adiposity cannot be excluded. Excess weight has been associated with altered inflammatory profiles, impaired small-airway function, and potentially differential responses to biologic therapy. Future studies incorporating metabolic and body-composition phenotyping are warranted to clarify these relationships.

Several limitations should be acknowledged. The retrospective and single-center design may limit generalizability. Incomplete availability of certain biomarkers, particularly FeNO, restricted the robustness of selected analyses. Sample sizes within biologic subgroups were modest, especially at longer follow-up time points, limiting statistical power for between-group comparisons. T2-high classification was based exclusively on baseline biomarker values, and misclassification cannot be excluded in patients with fluctuating inflammatory profiles. The absence of systematically recorded historical biomarker trajectories may have further contributed to classification uncertainty.

Biologic selection was guided by baseline clinical characteristics and inflammatory biomarkers, reflecting routine clinical decision-making. Consequently, confounding by indication and residual confounding are inherent to this observational design. Although multivariable adjustment was applied, observed differences in lung function trajectories should be interpreted as associative rather than causal. Furthermore, the absence of a non-biologic control group limits the ability to distinguish treatment-related effects from the natural course of disease or regression to the mean. However, withholding biologic therapy from eligible patients would not have been ethically feasible in routine practice.

A key observation of this study was the high prevalence of reduced FEF25–75 values across biologic groups. In this study, FEF25–75 was interpreted as a spirometric surrogate suggestive of small airways involvement, and changes in this parameter generally paralleled changes in FEV_1_. However, FEF25–75 should be interpreted cautiously, as it is effort-dependent, influenced by FVC, and is not a gold-standard measure of small airways disease. In addition, advanced small-airway assessments (including impulse oscillometry, nitrogen washout, RV/TLC, or CT air-trapping analysis) were not systematically available in this retrospective real-world cohort, which limits a more direct assessment of distal airway dysfunction.

Despite these limitations, this study provides detailed real-world phenotypic characterization, incorporates systematic assessment of small airways function, and offers comprehensive evaluation of treatment persistence and switching patterns. These findings contribute to a better understanding of biologic-treated severe asthma in routine clinical care and support the continued development of individualized, phenotype-driven management strategies.

## 5. Conclusions

In this real-world cohort of patients with severe asthma, biologic therapy was associated with stabilization or improvement of lung function, including spirometric indices suggestive of small airways involvement, despite long-standing disease and high baseline severity. Distinct phenotypic profiles were observed across biologic treatment groups, reflecting real-world precision medicine practice driven by dominant inflammatory traits rather than clearly separable baseline severity.

Anti-IL-5/IL-5R therapies were predominantly used in patients with eosinophilic, often steroid-dependent asthma with prominent small airways dysfunction, demonstrating gradual functional stabilization over time. Although heterogeneous temporal patterns in lung function trajectories were observed across biologic groups, no clear superiority of one biologic class was demonstrated. Observed differences likely reflect underlying phenotypic variation and biomarker-driven treatment selection in routine clinical practice.

Overall, these findings underscore the importance of integrating clinical characteristics, inflammatory biomarkers, and functional indices—particularly spirometric small-airway surrogates where advanced techniques are not available—when selecting and monitoring biologic therapy in severe asthma. Longitudinal real-world data and continued phenotypic reassessment remain essential to optimize treatment response, guide biologic switching, and advance personalized management strategies in severe asthma.

## Figures and Tables

**Table 1:** Baseline characteristics by biologic treatment group.

Variable	Benralizumab (n = 13)	Omalizumab (n = 10)	Mepolizumab (n = 30)	Tezepelumab (n = 34)	*p*-Value
Female sex, n (%)	10 (76.9)	8 (75.0)	23 (75.0)	26 (75.9)	0.999
Age (years)	59.3 ± 13.4	42.5 ± 24.8	60.5 ± 12.9	63.2 ± 11.3	0.287
BMI (kg/m^2^)	30.2 ± 6.1	30.0 ± 5.0	30.0 ± 5.1	30.7 ± 7.1	0.282
Asthma duration (years)	26.8 ± 15.9	13.5 ± 5.0	24.0 ± 21.0	15.2 ± 15.4	0.145
Total IgE (IU/mL)	491.8 ± 248.7	586.5 ± 556.5	730.1 ± 1419.3	166.5 ± 176.7	0.007
FeNO (ppb)	15.0 ± 14.1	20.5 ± 6.4	25.5 ± 6.0	12.6 ± 10.7	0.561
Blood eosinophils (cells/μL)	503.0 ± 592.0	203.0 ± 303.0	541.0 ± 311.0	181.0 ± 167.0	<0.001
FEV_1_ (% predicted)	87.0 ± 19.9	81.7 ± 33.6	94.9 ± 20.0	85.9 ± 19.4	0.395
FEV_1_/FVC (%)	78.1 ± 9.3	80.1 ± 9.2	77.4 ± 7.9	75.9 ± 8.8	0.493
FEV_1_/FVC < 70%	2 (15.0)	1 (10.0)	3 (10.0)	6 (17.6)	0.817
FEF25–75 (% predicted)	80.1 ± 43.2	81 ± 43.2	73.8 ± 23.5	70.8 ± 33.2	0.386
FEF25–75 < 70%	2 (15.4%)	3 (30.0%)	15 (50.0)	13 (38.2)	0.066
Never Smokers	10 (76.9)	8 (80.0)	20 (66.6)	27 (79.4)	0.076
T2-high phenotype	13 (100)	10 (100)	30 (100)	19 (55.6)	<0.001
ER visits, prior 12 months (mean ± SD)	0.38 ± 0.65	0.33 ± 0.58	0.47 ± 0.73	0.41 ± 0.66	0.912
Hospitalizations, prior 12 months (mean ± SD)	0.23 ± 0.44	0.20 ± 0.42	0.30 ± 0.60	0.26 ± 0.51	0.934
≥1 ER visit, n (%)	4 (30.8)	3 (33.3)	9 (33.3)	11 (34.5)	0.998
≥1 hospitalization, n (%)	3 (23.1)	2 (20.0)	6 (20.0)	7 (20.6)	0.996
OCS use, n (%)	5 (38.5)	3 (30.0)	10 (33.3)	11 (32.4)	0.964
Maintenance OCS daily dose at initiation (prednisolone-equivalent, mg/day), median (IQR)	7.5 (5–10)	5 (2.5–10)	7.5 (5–10)	7.5 (5–10)	0.818

Footnote: Continuous variables were compared using one-way ANOVA or Kruskal–Wallis test, as appropriate. Categorical variables were compared using chi-square test or Fisher’s exact test when expected cell counts were small. *p*-values are reported for overall between-group comparisons across biologic treatment classes. Maintenance OCS daily dose is reported among patients receiving maintenance OCS at initiation.

**Table 2:** Adjusted longitudinal mixed-effects model estimates for change in FEV_1_ (% predicted).

Biologic Therapy	Adjusted Mean Change (β)	95% Confidence Interval	*p*-Value
Benralizumab	+12.46	+1.63 to +19.29	0.020
Mepolizumab	+15.82	+8.35 to +23.64	<0.001
Omalizumab	+16.65	+1.58 to +31.71	0.030
Tezepelumab	+15.69	+6.52 to +24.87	<0.001

Footnotes: Estimates derived from multivariable linear mixed-effects models with patient-specific random intercepts and time modeled categorically (baseline, 6 months, 12 months). Models were adjusted for age, sex, body mass index, smoking status, baseline FEV_1_ % predicted, baseline blood eosinophil count, asthma disease duration, and maintenance oral corticosteroid use.

## Data Availability

The data presented in this study are available from the corresponding author upon reasonable request, due to privacy and ethical restrictions.

## Supplementary Materials

The following supporting information can be downloaded at: https://www.mdpi.com/article/10.3390/jpm16070362/s1, Supplementary File S1: Strobe Checklist; Table S1. Availability of post-bronchodilator spirometric assessments during follow-up; Table S2. Availability of baseline inflammatory biomarker measurements by biologic group; Table S3. Annualized exacerbation outcomes pre- and post-biologic therapy; Table S4. Sensitivity analysis subgroup defined by baseline eosinophils ≥300 cells/µL.
