# Supplementary material for: Real-World Phenotypic Profiles and Longitudinal Lung Function Outcomes in Severe Asthma Treated with Biologic Therapies
Source: J Pers Med. 2026 Jul 3;16(7):362. doi: 10.3390/jpm16070362 (PMC13412653; doi:10.3390/jpm16070362)
Supplement: Supplementary file 1 [file jpm-16-00362-s001.zip › Supplementary File S1. Strobe Checklist.pdf]

Supplementary File S1. STROBE Checklist

Study type: Retrospective observational cohort study
Manuscript title: *Real-World Phenotypic Profiles and Longitudinal Lung Function Outcomes in Severe Asthma Treated with Biologic Therapies*

Item	Recommendation	Reported in manuscript
**1(a)**	Indicate the study's design with a commonly used term in the title or the abstract	**Abstract (Methods)**: "retrospective observational study"; **Methods 2.1**: "real-world, observational study"
**1(b)**	Provide in the abstract an informative and balanced summary of what was done and what was found	**Abstract** (Background, Objective, Methods, Results, Conclusions)
**2**	Explain the scientific background and rationale for the investigation	**Introduction** (paras 1–4)
**3**	State specific objectives, including any prespecified hypotheses	**Introduction (final paragraph)**; **Methods 2.4 Study Outcomes**
**4**	Present key elements of study design early in the paper	**Methods 2.1 Study design and setting**
**5**	Describe the setting, locations, and relevant dates, including periods of recruitment, exposure, follow-up, and data collection	**Methods 2.1–2.2** (University of Thessaly Asthma Clinic, Greece; study period 2021–2025)
**6(a)**	Give eligibility criteria and the sources and methods of selection of participants. Describe methods of follow-up	**Methods 2.2 Study population**; **Methods 2.6 Lung function assessment**; **Methods 2.7 Treatment outcomes/persistence**
**6(b)**	For matched studies, give matching criteria and number of exposed/unexposed	**Not applicable** (no matching design used)
**7**	Clearly define all outcomes, exposures, predictors, potential confounders, and effect modifiers. Give diagnostic criteria, if applicable	**Methods 2.4 Study Outcomes**; **2.5 Data collection and variables**; **2.6 Lung function assessment**; severe asthma criteria in **2.2**
**8**	For each variable of interest, give sources of data and details of methods of assessment	**Methods 2.5** (clinical, biomarker variables); **2.6** (spirometry methods and timing)
**9**	Describe any efforts to address potential sources of bias	**Methods 2.8 Statistical analysis** (multivariable adjustment for confounding by indication; mixed-effects models); **Discussion—Limitations**
**10**	Explain how the study size was arrived at	**Methods 2.2** (all biologic-treated patients in clinic population during study period

Item	Recommendation	Reported in manuscript
		were included; annual clinic population ~800)
**11**	Explain how quantitative variables were handled in the analyses. If applicable, describe which groupings were chosen and why	**Methods 2.5** (T2-high/T2-low definitions; biomarker thresholds); **2.8** (continuous/categorical summaries; time modeled categorically)
**12(a)**	Describe all statistical methods, including those used to control for confounding	**Methods 2.8 Statistical analysis** (ANOVA/Kruskal–Wallis, chi-square/Fisher, mixed-effects models, covariate adjustment)
**12(b)**	Describe any methods used to examine subgroups and interactions	**Methods 2.8** (time × biologic interaction; sensitivity analyses incl. T2-high/eosinophils ≥300 cells/µL)
**12(c)**	Explain how missing data were addressed	**Methods 2.8** (mixed-effects models under MAR; all available observations used; no listwise deletion)
**12(d)**	If applicable, explain how loss to follow-up was addressed	**Methods 2.7** (censoring definitions; last follow-up/last contact); **Methods 2.6** and **Results 3.6** (availability by time point)
**12(e)**	Describe any sensitivity analyses	**Methods 2.8** (complete-case and T2-high/eosinophilic subgroup); **Results 3.7** (sensitivity analysis results)
**13(a)**	Report numbers of individuals at each stage of study, e.g., potentially eligible, included, followed up, analyzed	**Results 3.1** (87 included from 800 clinic population); **Results 3.6** and **Supplementary Table S1** (follow-up availability by time point)
**13(b)**	Give reasons for non-participation at each stage	**Partially addressed**: discontinuation and switching reasons in **Results 3.9–3.10**; no formal non-participation flowchart (all eligible biologic-treated patients included)
**13(c)**	Consider use of a flow diagram	**Not included** (optional; could be added if desired)
**14(a)**	Give characteristics of study participants and information on exposures and potential confounders	**Results 3.2–3.5**; **Table 1**
**14(b)**	Indicate number of participants with missing data for each variable of interest	**Partially addressed**: FeNO “when available” and follow-up availability in **Supplementary Table S1**; could be expanded for each variable if required
**14(c)**	Summarize follow-up time (e.g., average and total amount)	**Methods 2.6** (median follow-up defined/calculated); **Methods 2.7** (follow-

Item	Recommendation	Reported in manuscript
		up linked to biologic exposure); partial reporting in Results
**15**	Report numbers of outcome events or summary measures over time	**Results 3.4, 3.6, 3.7, 3.9, 3.10**; **Table 2**; Supplementary tables
**16(a)**	Give unadjusted estimates and, if applicable, confounder-adjusted estimates and their precision (e.g., 95% CI)	**Results 3.6** and **Table 2** (adjusted β, 95% CI, p-values); baseline group comparisons in **Table 1**
**16(b)**	Report category boundaries when continuous variables were categorized	**Methods 2.5** (T2-high definition thresholds, FeNO, eosinophils)
**16(c)**	If relevant, consider translating estimates of relative risk into absolute risk for a meaningful time period	**Not applicable** (no risk ratio design/endpoints)
**17**	Report other analyses done (subgroups, interactions, sensitivity analyses)	**Methods 2.8** and **Results 3.6–3.7** (interaction, sensitivity analyses, subgroup interpretation)
**18**	Summarize key results with reference to study objectives	**Discussion opening paragraphs**; **Conclusions**
**19**	Discuss limitations of the study, taking into account sources of potential bias or imprecision	**Discussion—Limitations paragraphs** (retrospective single-center design, subgroup size, biomarker availability, confounding by indication, no control group, FEF25–75 limitations)
**20**	Give a cautious overall interpretation of results considering objectives, limitations, multiplicity of analyses, results from similar studies, and other evidence	**Discussion** (interpretive paragraphs on heterogeneity, non-causal inference, real-world prescribing)
**21**	Discuss the generalizability (external validity) of the study results	**Discussion—Limitations/interpretation** (single-center tertiary clinic; generalizability acknowledged)
**22**	Give the source of funding and the role of the funders	**Funding** section ("This study received no funding")
