# Supplementary material for: Real-World Phenotypic Profiles and Longitudinal Lung Function Outcomes in Severe Asthma Treated with Biologic Therapies
Source: J Pers Med. 2026 Jul 3;16(7):362. doi: 10.3390/jpm16070362 (PMC13412653; doi:10.3390/jpm16070362)
Supplement: Supplementary file 1 [file jpm-16-00362-s001.zip › Supplementary Tables.pdf]

Supplementary Table S1. Availability of post-bronchodilator spirometric assessments during follow-up.

Follow-up Time Point	Overall (n = 87)	Benralizumab (n = 13)	Omalizumab (n = 10)	Mepolizumab (n = 30)	Tezepelumab (n = 34)
Baseline	87	13	10	30	34
6 months	87	13	10	30	34
12 months	77	13	10	30	24
24-36 months	42	11	8	23	0

Footnote: Post-bronchodilator spirometry was performed at baseline and during routine clinical follow-up. The 24–36-month time point includes assessments conducted between 24 and 36 months after biologic initiation. The number of available spirometric assessments decreased over time due to differential follow-up duration and discontinuation of the index biologic (15/87, 17.2%, overall). Patients continued to undergo spirometric monitoring in routine care; however, analyses are presented according to the availability of measurements within the index-biologic follow-up window. Tezepelumab first became available in Greece in September 2024, which explains the absence of 24–36-month follow-up data for this group.

Supplementary Table S2. Availability of baseline inflammatory biomarker measurements by biologic group

Biomarker	Overall (n = 87)	Benralizumab (n = 13)	Omalizumab (n = 10)	Mepolizumab (n = 30)	Tezepelumab (n = 34)
Blood eosinophil count (cells/μL)	87	13	10	30	34
Total serum IgE (IU/mL)	87	13	10	30	34
FeNO (ppb)	58	10	7	23	18

Footnote: Biomarker measurements were obtained at baseline prior to biologic initiation as part of routine clinical assessment. FeNO availability was dependent on equipment access and clinical indication. The median follow-up duration for the overall cohort was 30 months (interquartile range 26–34), with comparable follow-up durations across biologic treatment groups.

Supplementary Table S3. Annualized exacerbation outcomes pre- and post-biologic therapy.

**Outcome**	**Pre-biologic (12 months prior)**	**Post-biologic (follow-up)**	**Mean Difference (95% CI)**	**p-value**
Annualized exacerbation rate (events/patient-year)	1.8 ± 1.2	0.7 ± 0.9	−1.1 (−1.4 to −0.8)	<0.001
Exacerbations requiring systemic corticosteroids	1.4 ± 1.0	0.5 ± 0.7	−0.9 (−1.2 to −0.6)	<0.001
Emergency department visits	0.42 ± 0.66	0.15 ± 0.38	−0.27 (−0.41 to −0.13)	0.002
Hospitalizations	0.26 ± 0.51	0.08 ± 0.29	−0.18 (−0.30 to −0.06)	0.010

Supplementary Table S4. Sensitivity analysis subgroup defined by baseline eosinophils ≥300 cells/µL.

**Biologic group**	**n with eos ≥300**	**n with paired baseline + 12-month FEV_1**
Benralizumab	6	6
Omalizumab	3	3
Mepolizumab	19	19
Tezepelumab	4	4
**Total**	**32**	**32**

Footnote: The eosinophil ≥300 cells/µL subgroup was used as a stricter biomarker-based sensitivity analysis to complement the primary T2-high classification. This sensitivity analysis was restricted to longitudinal spirometric outcomes (paired baseline and 12-month FEV_1) and was interpreted as supportive/exploratory.
